# Motor, epileptic, and developmental phenotypes in genetic disorders affecting G protein coupled receptors-cAMP signaling

**DOI:** 10.3389/fneur.2022.886751

**Published:** 2022-08-08

**Authors:** Serena Galosi, Luca Pollini, Maria Novelli, Katerina Bernardi, Martina Di Rocco, Simone Martinelli, Vincenzo Leuzzi

**Affiliations:** ^1^Department Human Neuroscience, Sapienza University, Rome, Italy; ^2^Department of Oncology and Molecular Medicine, Istituto Superiore di Sanità, Rome, Italy

**Keywords:** *GNAO1* encephalopathy, *GNB1*, *ADCY5*, *PDE2A*, *PDE10A*, cAMP, GPCR (G protein coupled receptor)

## Abstract

Over the last years, a constantly increasing number of genetic diseases associated with epilepsy and movement disorders have been recognized. An emerging group of conditions in this field is represented by genetic disorders affecting G-protein-coupled receptors (GPCRs)–cAMP signaling. This group of postsynaptic disorders includes genes encoding for proteins highly expressed in the central nervous system and involved in GPCR signal transduction and cAMP production (e.g., *GNAO1, GNB1, ADCY5, GNAL, PDE2A, PDE10A*, and *HPCA* genes*)*. While the clinical phenotype associated with *ADCY5* and *GNAL* is characterized by movement disorder in the absence of epilepsy, *GNAO1, GNB1, PDE2A, PDE10A*, and *HPCA* have a broader clinical phenotype, encompassing movement disorder, epilepsy, and neurodevelopmental disorders. We aimed to provide a comprehensive phenotypical characterization of genetic disorders affecting the cAMP signaling pathway, presenting with both movement disorders and epilepsy. Thus, we reviewed clinical features and genetic data of 203 patients from the literature with GNAO1, GNB1, PDE2A, PDE10A, and HPCA deficiencies. Furthermore, we delineated genotype–phenotype correlation in GNAO1 and GNB1 deficiency. This group of disorders presents with a highly recognizable clinical phenotype combining distinctive motor, epileptic, and neurodevelopmental features. A severe hyperkinetic movement disorder with potential life-threatening exacerbations and high susceptibility to a wide range of triggers is the clinical signature of the whole group of disorders. The existence of a distinctive clinical phenotype prompting diagnostic suspicion and early detection has relevant implications for clinical and therapeutic management. Studies are ongoing to clarify the pathophysiology of these rare postsynaptic disorders and start to design disease-specific treatments.

## Introduction

A significant number of genes associated with paroxysmal and non-paroxysmal movement disorders (MDs) and epilepsy have been recognized in recent years, shedding light on the biological substrates and pathways involved in these conditions.

Recently described genes in this field encode for proteins involved in postsynaptic signaling pathways downstream to G-protein-coupled receptors (GPCRs), which are ubiquitously expressed in the central nervous system (CNS) and highly enriched in striatal medium spiny neurons (MSNs) (1, 2). GPCRs control responses to a wide array of sensory stimuli, including light and odorants, and non-sensory stimuli, including neurotransmitters and hormones. Signal transduction *via* GPCRs relies primarily upon the activation of heterotrimeric G-proteins, which consist of an α-subunit that binds and hydrolyzes GTP and a βγ heterodimer (3). GTP binding can induce an allosteric transition leading to βγ release which, in turn, enables Gα and Gβγ signaling to their multiple downstream effectors (4). The variety and expression pattern of individual G-protein subunits define unique GPCR properties in a cell-context-specific manner.

**Figure 1:**
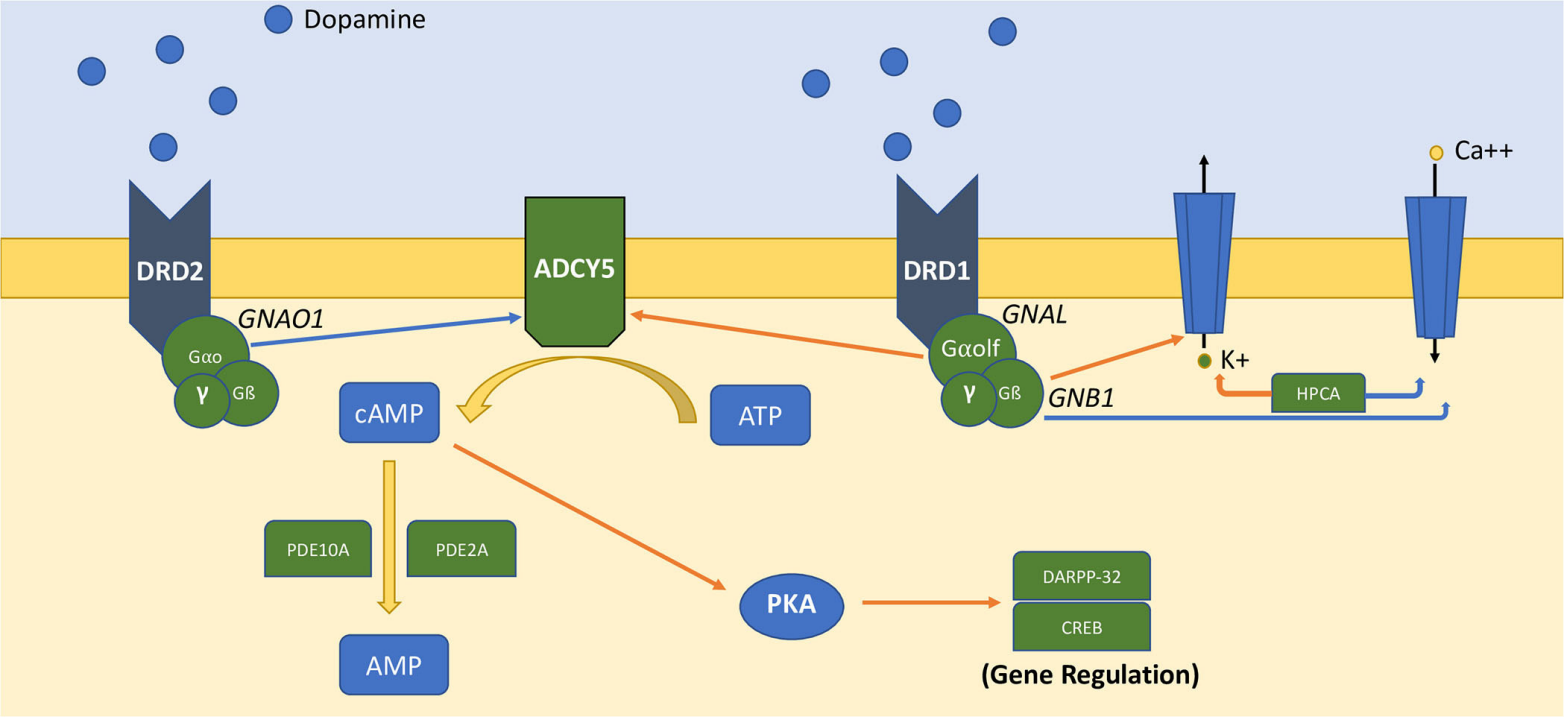
Simplified representation of DA-dependent GPCRs–cAMP signaling pathway in medium spiny neurons. Orange arrows represent activation signals, and blue arrows represent inhibitory signals. ATP is converted to AMP by adenyl-cyclase-5 (ADCY5), which is regulated by Gαo (*GNAO1*) and Gαolf (*GNAL*). Gß subunit (*GNB1*) and hippocalcin (HPCA) regulate the activity of potassium and calcium channels. The generated cAMP propagates downstream signaling via cAMP-binding proteins. cAMP is converted to AMP by phosphodiesterase activity (PDE10A, PDE2A).

Genes discovered in this pathway and associated with neurological disorders encode transducer molecules or components of the GPCR machinery (i.e., *GNAO1, GNB1, GNAL, GPR88*), or proteins controlling the synthesis and hydrolysis of the second messenger cyclic adenosine monophosphate (cAMP) (*ADCY5, PDE10A, PDE2A*). Although the precise functions are still largely unknown, *HPCA* (encoding for hippocalcin) is a calcium sensor associated with the plasma membrane that influences the activity of potassium and calcium channels and could be implied in the modulation of dopamine post signaling in striatal neurons (1, 2). Figure 1 represents the organization of this signaling pathway in MSNs (Figure 1). The exact role of these proteins in the pathophysiology of MDs and the functional impact of pathogenic variants have only begun to be explored in preclinical models. For a comprehensive review on this topic, see Golzales-Latapi et al. (2).

*GNAL* (OMIM 139312) and *ADCY5* (OMIM 600293) genes encode for G_olf_ and adenylyl cyclase type 5 (AC5), the main AC isoform expressed in the striatum, respectively, which are directly involved in the GPCR–cAMP signaling cascade mediated by the activation of dopamine receptor 1 (D1R) and adenosine receptor 2A (A2AR) in MSNs (5).

G_olf_ is responsible for coupling D1R and A2AR stimulation to the activation of ADCY5, causing increased intracellular cAMP levels, which, in turn, enhances neuronal activity (6). Pathogenic variants in *GNAL* and *ADCY5* may manifest through either autosomal dominant or recessive modes of inheritance. Most *GNAL* mutations are heterozygous missense, nonsense, or frameshift variants with a clear loss-of-function (LOF) effect (7, 8) leading to reduced cAMP levels. In contrast, most disease-causing *ADCY5* variants are gain-of-function (GOF) defects causing increased cAMP production (2). These findings suggest a complex scenario in which both increased and decreased intraneuronal cAMP levels may underlie the pathogenesis of MDs.

The critical role of the GPCRs–cAMP signaling pathway in the pathophysiology of MDs has been further highlighted by the identification of disease-causing variants in *PDE10A* (OMIM 610652) and *PDE2A* (OMIM 602658). These genes encode two cyclic nucleotide phosphodiesterases (PDEs) highly expressed in MSNs and critically involved in modulating dopaminergic and adenosinergic responses through degradation of intracellular cAMP and cGMP (9–13). While both homozygous and heterozygous LOF mutations have been reported in *PDE10*, only biallelic changes have been identified in *PDE2A* so far.

*GNAO1* (OMIM 139311) and *GNB1* (OMIM 139380) encode proteins that are components of the GPCR machinery, respectively the alpha subunit (Gαo) and the beta-1 (Gß1) subunit of the inhibitory heterotrimeric Go-protein complex (3). *GNAO1* and *GNB1* are co-expressed in the cerebral cortex, hippocampus, and striatum where they are involved in transducing signals downstream to several GPCRs and in the regulation of AC activity.

*GNAO1* modulates inhibitory signaling from several GPCRs, including GABA-B, dopamine D2, α2A adrenergic, and adenosine A1, regulating neuronal excitability and neurotransmission (3), and controls neurodevelopment (14). In the brain, Go controls the synthesis of cAMP, directly prevents neurotransmitter release, inhibits N-type and P/Q-type calcium channels, and activates G-protein-coupled inward rectifying potassium (GIRK) channels. Regarding striatal pathways, Gαo activity influences the excitability of neurons of the indirect (inhibitory MSNs, iMSNs) and direct pathways (dMSNs) by tuning inputs from dopamine D2 and adenosine A2A receptors, with crucial effects on movement control (15). In dMSNs, Gao affects both the efficacy (defined as the power to produce an effect) and potency (defined as the capacity to produce strong physiological or chemical effects) of responses to dopamine while only modulating adenosine efficacy. Instead, in iMSNs, Gao affects both efficacy and potency of responses to adenosine while only modulating dopamine efficacy. Taken together, these data indicate that Gao plays a pivotal role in controlling the potency and efficacy of stimulatory neuromodulation while only affecting the efficacy of inhibitory inputs in both populations of striatal neurons (15). A preclinical model of Gao defect revealed a different motor impairment as a result of knocking out Gao in dMSN or iMSN. In the first case, the acquisition and retention of motor skills were mainly affected. In the second one, dystonia and profound coordination deficits were observed (15).

Aberrant cAMP synthesis was originally proposed as the main pathogenic mechanism of the disease in *GNAO1* disorders, with LOF and GOF alleles that appeared to be primarily associated with epilepsy and MD, respectively (16). These data apparently contradict the original findings from Nakamura et al. (17) suggesting a LOF behavior of *GNAO1* variants on Gα_o_-mediated signaling, regardless of the associated clinical presentation. In a recently published and elegant study performed by Muntean and coworkers, *GNAO1* variants were shown to disturb Gαo function in a cell-type-specific manner *via* a combination of LOF and dominant-negative mechanisms that are not mutually exclusive (15). More recently, a strong hypomorphic effect or a complete LOF behavior has been confirmed in genetically modified nematodes harboring *GNAO1* pathogenic variants, leading to excessive neurotransmitter release by different classes of motor neurons (18, 19). Of note, the observed phenotype was shown to be ameliorated by caffeine *via* adenosine receptor antagonism (18).

*GNB1* encodes Gβ1, the third component of the heterotrimeric G-protein complex. Upon receptor activation, Gβ1 dissociates from the Gα subunit and, together with the Gγ subunit, activates downstream signaling pathways, leading to inhibition of presynaptic voltage-gated calcium (i.e., Cav2.1 and Cav2.2) and potassium channels, with effects on neurotransmitter release (20, 21). Gβ1 interacts also with Gα_olf_ in striatal neurons, and Lohmann et al. showed that pathogenic *GNB1* variants might reduce association with Gα_olf_ thus reducing coupling to D1R (22). The functional impact of dominant *GNB1* mutations is still debated.

The clinical spectrum associated to this group of conditions ranges from developmental epileptic encephalopathy with severe early-onset movement disorder to isolated paroxysmal and/or non-paroxysmal movement disorders.

Clinical studies, preclinical models, and systems biology analysis helped to understand the relevance of these genes encoding postsynaptic signaling proteins in different subtypes of striatal cells to the pathogenesis of hyperkinetic MDs (2). The clinical features other than MD and the pathophysiology of epilepsy and developmental issues in these disorders have been less investigated and remain poorly understood.

Here, we reviewed the clinical phenotypes and mutational spectrum associated with GPCRs signaling disorders focusing on the conditions of this group presenting with epilepsy, movement disorders, and neurodevelopmental disorders. We aimed to verify the presence of common clinical features and characterize the core clinical phenotype of this group of severe early-onset genetic neurological disorders. Considering their clinical severity and susceptibility and precipitation with specific triggers, this in-depth clinical characterization has implications for timely diagnosis, management, and therapeutic strategies.

## Materials and methods

A comprehensive search of the medical literature (PubMed, Medline, Cochrane CENTRAL, Google Scholar) was conducted to identify papers reporting patients with pathogenic or likely pathogenic variants in *GNAO1, GNB1, ADCY5, GNAL PDE2A, PDE10A, HPCA. “GNAO1,” “GNB1,” ”ADCY5,” ”GNAL,” “PDE2A,” “PDE10A,”* and “*HPCA”* were used as search terms. As possible limitations of our search, we declare to have selected only English-written articles. It is possible that by doing so, some information included in non-English-written papers and useful to further delineate the clinical phenotype of these rare disorders could have been missed. Furthermore, variants were not independently re-evaluated as they were already judged as pathogenic or likely pathogenic according to ACMG criteria or reported in multiple patients with similar clinical presentations. We selected and reviewed cases with pathogenic or likely pathogenic variants according to ACMG criteria for which clinical, neuroradiological, neurophysiological, and genetic data were available. We focused on disorders with MDs, epilepsy, and neurodevelopmental disorders in their clinical spectrum.

## Results

**Figure 2:**
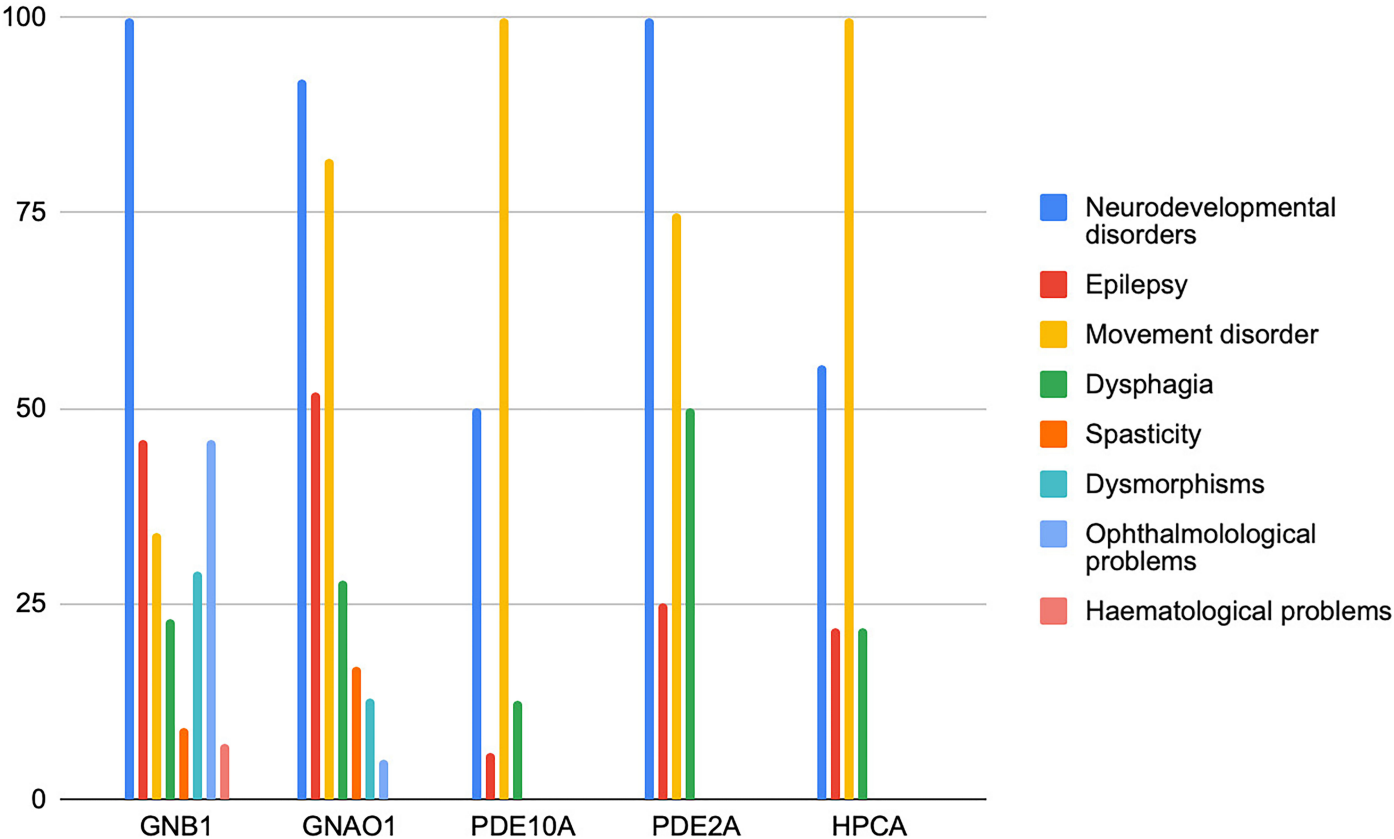
Distribution of the main clinical features of genetic disorders affecting the GPCRs–cAMP signaling pathway. The frequency of symptoms is expressed in the percentage of patients.

**Figure 3:**
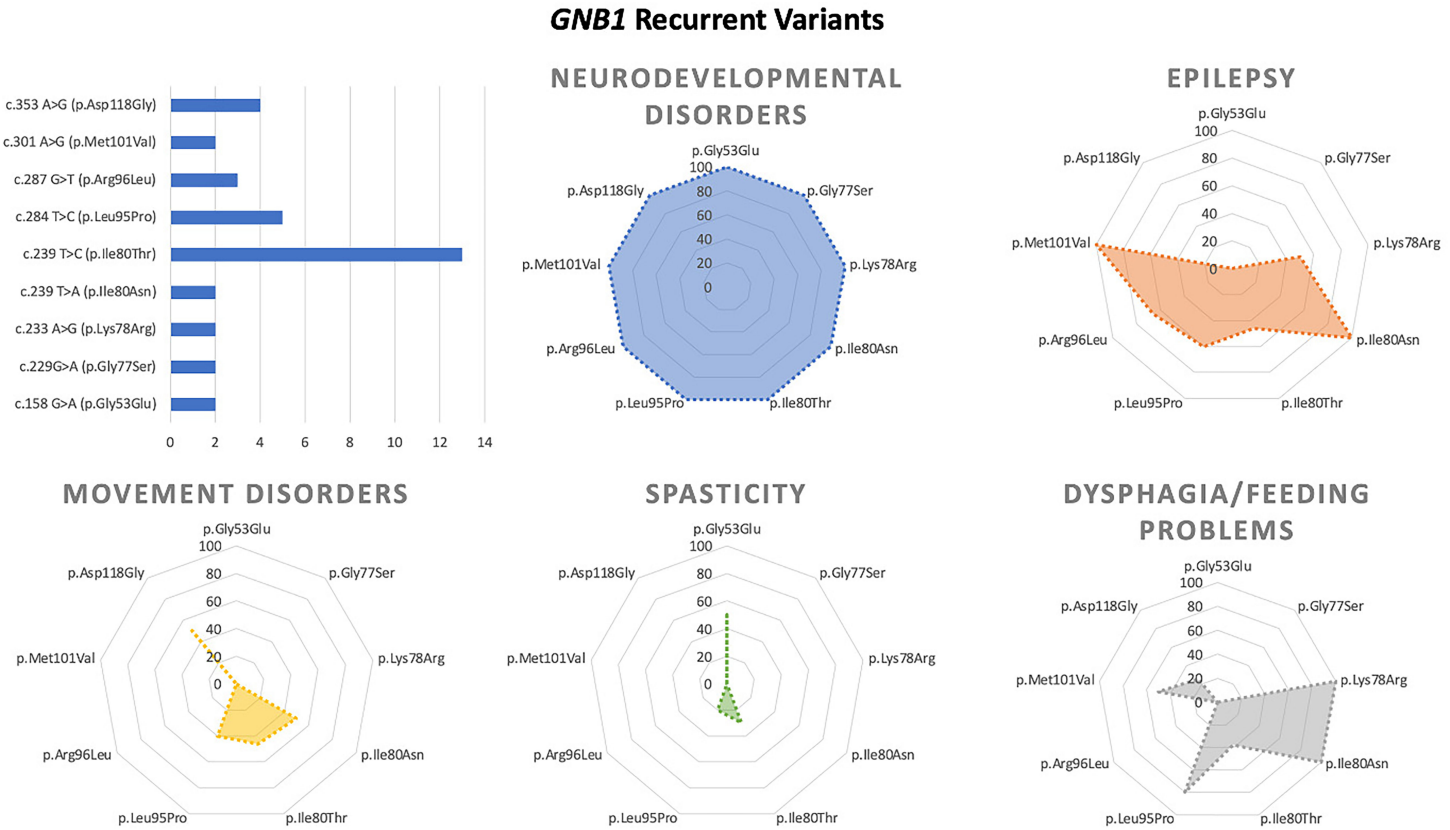
Main genotype–phenotype correlations in GNB1 deficiency.

**Figure 4:**
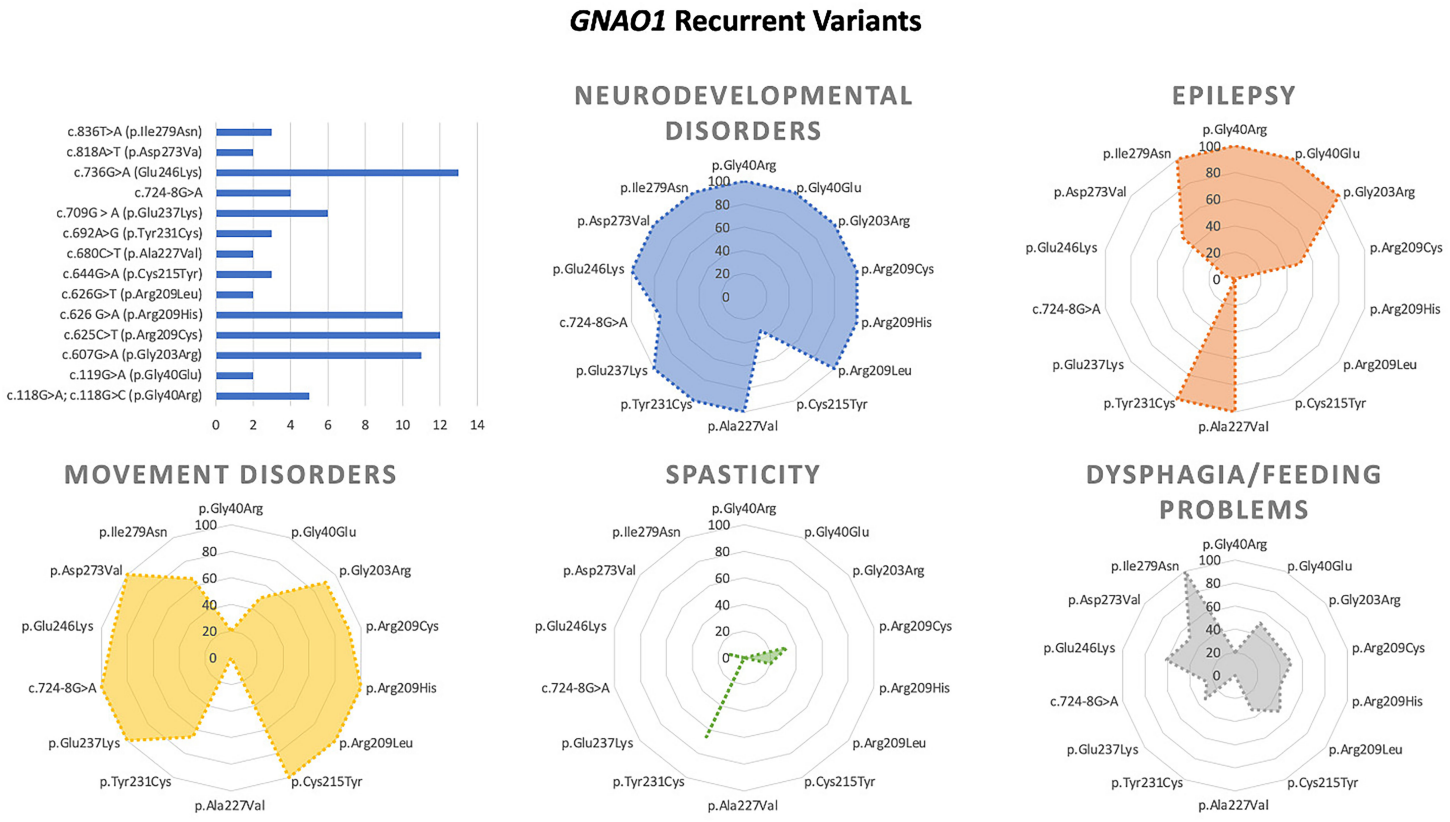
Main genotype–phenotype correlations in GNAO1 deficiency.

We collected and reviewed 74 articles (17 for *GNB1*, 46 for *GNAO1*, 6 for *PDE10A*, 2 for *PDE2A*, and 3 for HCPCA) describing motor, epileptic, and developmental phenotype of patients with genetic disorders of GPCRs–cAMP signaling pathway. Six additional papers on *GNAO1* were excluded (four because of insufficient information and two because of the presence of concomitant mutations in other genes). Finally, to the scope of this review 203 cases from 72 papers published up to May 2022 were selected. Figure 2 represents the main clinical features associated with the above-mentioned genes. Characteristics of GNAO1 and GNB1 deficiency were further evaluated to assess for a genotype–phenotype correlation (Figures 3, 4).

### GNB1

*De novo* mutations in *GNB1* cause an autosomal dominant neurodevelopmental disorder (MIM #616973), which may present as isolated or as part of a wide range of both neurological and non-neurological features (22). This gene was first associated with human disease in 2016 (23), and to date, approximately 64 patients carrying 38 different pathogenetic variants have been reported (22–38).

#### Neurodevelopmental phenotype

Neurodevelopmental delay is described as severe in most individuals and is often the presenting symptom. Motor control is usually limited to the head or trunk, with only a dozen of patients achieving assisted or independent walking from 3 years of age or later in life (23, 25, 27, 29, 30) and very rare individuals showing normal motor development (22, 27, 29). Language is severely impaired, even compared to motor achievements. Most patients are non-verbal, and a small number of them can only use single words (23–25, 27, 29). A moderate to severe intellectual disability, variably associated with other neurodevelopmental disorders such as ADHD and Autism spectrum disorders, has been reported in most of the patients, whereas only sporadic cases showed mild intellectual disability (29, 30).

#### Epilepsy

Infancy or childhood-onset epilepsy has been described in 30 individuals and is one of the most challenging issues in GNB1 deficiency. Substitutions located in exons 6 and 7, in particular the ones at residue Ile^80^ (p.Ile80Thr and p.Ile80Asn) and Leu^95^ (p.Leu95Pro), have been associated to infantile-onset seizures. Infantile spasms and hypsarrhythmia represent a common presentation in these patients (23, 28, 31, 32). Other infantile epileptic presentations include sporadic cases of tonic-clonic (one patient), tonic (two patients), and myoclonic seizures (two patients), usually poorly controlled by antiepileptic drugs (23). Febrile status epilepticus anticipating non-febrile seizures has also been reported (23). Patients with childhood-onset epilepsy show a wide range of seizure types, including motor (tonic-clonic and myoclonic) and non-motor seizures with impaired awareness (described as absences or staring spells) (23, 27). Epileptiform discharges are generally multifocal or, less frequently, focal or generalized. In many cases, epilepsy is drug-resistant or controlled by a combination of at least two drugs.

#### Movement disorder

Movement disorder is frequent in patients with *GNB1* pathogenic variants (23 individuals), usually dystonia and/or ataxia. Dystonia has been reported in 12 patients and was found to be more commonly associated with the p.Ile80Thr, p.Leu95Pro, p.Asp118Tyr, and p.Asp118Gly substitutions (22–25, 28, 30, 34). Myoclonus with dystonia has been reported in two patients (23, 29), suggesting a myoclonus–dystonia phenotype. Non-epileptic “twitches,” presumably myoclonus, have been reported in one patient (27). Ataxia has been observed in five patients with different pathogenetic variants (22). A combination of chorea and athetosis was described in three cases (22, 26, 27). Stereotypies such as body rolling and hand stereotypies were reported in four patients (23, 25–27), and tics (vocals and unspecified) in two (23, 27). A single patient with bradykinesia has been described (27). Episodic exacerbations of MD and status dystonicus have been reported in three patients [Galosi et al., 2022 (in press); (27, 28)]. Spasticity (either as quadriplegia or diplegia) was reported in six patients (27, 28).

Only three reports about pharmacological treatment in patients with GNB1 variants are available, and no drugs have been reported to dramatically improve movement disorder. Levodopa administration was not effective in one patient (24, 30). Deep brain stimulation (DBS) improved dystonia in two patients (30, 34).

#### Other features

Cortical blindness (4 patients) or oculomotor abnormalities including nystagmus (18 patients), strabismus (7 patients), and ophthalmoplegia (4 patients) have been described in nearly half of the GNB1 patients reported in the literature (22, 23, 27, 28).

A normal head circumference was seen in most of the patients (23, 27); macrocephaly and microcephaly were reported, respectively, in five and one cases. Cleft palate (often associated with the p.Leu95Pro variant) (31), growth delay, and other non-specific facial dysmorphisms may be part of the clinical spectrum. Dysphagia and feeding difficulties are frequent in GNB1 (15 pts) (23, 27, 28) leading, in severe cases, to tube feeding (23, 27). Hematological issues, such as cutaneous mastocytosis and acute lymphoblastic leukemia, have been reported in four and one patients, respectively (25–27). Three out of four individuals with cutaneous mastocytosis harbored the p.Ile80Thr substitution (26, 27).

Brain MRI findings range from normal to non-specific findings, such as white matter abnormalities (white matter hyperintensities or abnormal myelination) (10 patients), cerebellar hypoplasia (4 patients), generalized cortical atrophy and/or increased ventricular spaces (4 patients), and abnormalities of cortical gyri (3 patients) (22, 23, 25, 27, 28).

Among recurrent variants, the p.Asp118Gly substitution has been associated with neurodevelopmental disorder and dystonia without epilepsy, whereas the p.Ile80Thr and p.Leu95Pro changes are associated with both epilepsy and movement disorder.

### GNAO1

Dominant *GNAO1* mutations were first associated with human disease in 2013 (17) in a small cohort of patients with epileptic encephalopathy and the development of dyskinetic movement disorders in a subset of affected individuals. Three years later, Saitsu and colleagues first recognized the phenotype most frequently associated to this gene, namely, involuntary movement disorder and severe developmental delay with or without seizures (39).

To date, two main GNAO1-related disorders are reported in OMIM: early infantile-onset epileptic encephalopathy (EIEE17) (MIM#615473) and neurodevelopmental disorder with involuntary movements (NEDIM) (MIM#617493). Despite the present classification, the clinical practice is a variety of overlapping phenotypes with a small number of patients presenting isolated MD or epileptic manifestations. Thirty-nine patients show a mixed phenotype with movement disorder and epilepsy, 52 display isolated movement disorder, 16 experience epilepsy without movement disorder, and 3 have an unspecified neurodevelopmental disorder.

According to ClinVar (https://www.ncbi.nlm.nih.gov/clinvar/), 60 pathogenic/likely pathogenic variants have been reported in 111 patients. The vast majority of them are missense changes affecting more than 40 highly conserved residues. Approximately, half of the affected subjects harbor mutations affecting residues Gly^203^ (p.Gly203Arg), Arg^209^ (p.Arg209Cys/His/Gly), and Glu^246^ (p.Glu246Lys). Recent structural and functional data indicated that these and other *GNAO1* mutations variably affect Gα- and Gβγ-mediated signaling (15, 16, 19). Seventeen pathogenic variants recur in more than one patient.

#### Neurodevelopmental phenotype

Most patients presented with hypotonia (84/111 patients) and early developmental delay with significant impairment in both motor and language areas (102/111 patients).

#### Epilepsy

Epilepsy has been described in 58 individuals (nearly 50% of patients), with onset during the neonatal period (29 patients), infancy (18 patients), or childhood (8 patients) and outcome ranging from severe early-onset cases to milder forms.

Severe forms include developmental and epileptic encephalopathies (DEE) manifesting with infantile spasms or epilepsy of infancy with migrating focal seizures (EIFMS) (17). The p.Gly203Arg substitution appears to be the variant most frequently associated to early-onset epilepsy and movement disorder (40). Epilepsy in these patients is resistant to multiple medications. Other patients may manifest generalized and focal epilepsies at different ages (41). Patients carrying the c.625C>T (p.Arg209Cys) transition have childhood-onset epilepsy (3–12 years), with a high rate of generalized seizures and good response to treatment.

Overall, the highest rate of drug resistance was found in early-onset forms, while later-onset forms are usually better controlled by antiepileptic therapy.

The electroencephalographic abnormalities include hypsarrhythmia and burst suppression in early-onset forms, and focal and multifocal discharges in later presentations. Slow abnormalities have also been reported in several patients, even in the absence of epileptic manifestations.

#### Movement disorder

Movement disorder with onset during infancy or childhood (1 neonatal onset, 50 infantile onset, 13 childhood onset,) represents the core symptom (92 patients, 83% of cases) in patients with *GNAO1* variants. Chorea, athetosis, ballism, and dystonia, with a high impact on motor functioning, are the most commonly associated MDs (42).

Dystonia is reported in 63 patients, while chorea, athetosis, and ballism (often coexisting) are described in 52 patients. Moreover, almost constant is the finding of dyskinesia, reported in 37 patients, involving the orofacial district in one-third of patients (11 patients). More than one-third of patients with MD (34/87) experienced severe exacerbations. Specific variants appear to be more frequently associated with the occurrence of exacerbations: c.625C>T (p.Arg209Cys), c.736G>A (p.Glu246Lys), and c.709G>A (p.Glu237Lys). In these patients, the hyperkinetic movement disorder seems to be more disabling and potentially life-threatening, leading to a drug-resistant dystonic–dyskinetic state requiring surgical treatment (DBS and/or pallidotomy). Tetrabenazine appears to be the most used and effective pharmacological treatment, although sporadic cases of response to other drugs have been reported (risperidone, levetiracetam). In a minority of patients, myoclonus, ataxia, tremor [resting tremor in two patients (43, 44), not specified tremor in two patients (45, 46), tongue tremor in one patient] and parkinsonian features have been reported.

#### Other features

Dysphagia, often described, could lead in severe cases to tube feeding. Finally, MRI brain findings range from normal (59 patients) to different non-specific abnormalities, such as cerebral atrophy, abnormalities of myelination, or basal ganglia atrophy. In some cases, repeated MRI shows a lesional progression, suggesting a degenerative course.

### ADCY5

The constellation of neurological disorders associated with ADCY5 mutations includes conditions of variable severity, ranging from severe early-onset neurodevelopmental disorder with dyskinesia to familial dyskinesia with facial myokymia (FDFM). Complex combinations of paroxysmal and persistent MDs are possible, including chorea, dystonia, tremor, myoclonus, myokymia, and plegic attacks. Day and nighttime episodes (47) and facial dyskinesia are clue features to the diagnosis. Additional interictal features include axial and oral hypotonia, gaze abnormalities, spasticity, dysarthria, learning issues, and ADHD. Thus, far epilepsy has not been reported as a part of the phenotype. Nearly 119 cases have been described with only three patients reported with confirmed (one patient) (48) or suspected epileptic episodes (two patients) (49).

### PDE10A

Different studies described mutations in *PDE10A* causing childhood-onset chorea. Both heterozygous and compound heterozygous/homozygous mutations have been reported, with the latter showing an earlier onset of symptoms (8, 50). Based on OMIM classification, infantile-onset dyskinesia (MIM #616921) and striatal degeneration (MIM #616922) are associated with recessive and dominant modes of inheritance, respectively.

Diggle et al. described two different consanguineous families with biallelic mutations in *PDE10A*, c.320A>G (p.Tyr107Cys) and c.346G>C (p.Ala116Pro), both affecting exon 4. These individuals presented within infancy (mean age of 3 months) with axial hypotonia, dysarthria, and hyperkinetic movement disorder characterized by dyskinesia of the limbs, trunk, and face (50).

The c.320A>G (p.Tyr107Cys) variant was associated with orofacial dyskinesia, and drooling, generalized developmental delay but no cognitive impairment. Symptoms were less severe in older individuals than in younger ones.

No epilepsy was reported, and MRI, when done, was normal.

Two brothers carrying the c.346G>C (p.Ala116Pro) variant presented with developmental delay and at 7 years of age were able to speak single words. The second-born was more severely affected, and he was fed *via* a gastrostomy tube and presented focal epilepsy from 3.5 years of age. EEG revealed arrhythmic delta activity without focal epileptiform discharges, and treatment with carbamazepine had been effective.

Three *de novo PDE10A* mutations [c.898T>C (p.Phe300Leu), c.1000T>C (p.Phe334Leu), and c.1001T>G (p.F334C)] (9, 51–53) have been associated with childhood-onset chorea (5 to 10 years of age) with normal cognitive development and no epilepsy.

Interestingly, patients harboring dominant variant showed the presence of bilateral striatal abnormalities on brain MRI (9, 51, 52, 54).

### PDE2A

Biallelic *PDE2A* mutations cause a neurodevelopmental disorder with paroxysmal dyskinesia or seizures (MIM #619150). Four affected individuals have been described so far (9, 10). Interestingly, an intra-familiar phenotypic variability was evident in two siblings, with c.1180C>T predicting the formation of a premature stop codon (p.Gln394^*^), with one mainly suffering from epilepsy and the other from dystonia (12). All patients present intellectual disability or developmental delay.

MD is characterized by childhood-onset paroxysmal dyskinesia, with different triggers including emotional stress, sudden movements, or sudden sensorial stimuli. Episodes are usually brief (< 1 min) but frequent, until 100 episodes/day. Two patients developed sustained chorea-dystonia. Two cases showed persistent truncal hypotonia (12). Deep brain stimulation reduced the frequency and intensity of dyskinetic attacks in one case (11).

Infantile epilepsy with spasms and tonic seizures was reported in a single patient, resistant to multiple antiepileptic drugs, ketogenic diet, and vagus nerve stimulation (11). Ictal EEG recording showed epileptic spasms and right frontal seizures. The administration of vigabatrin and prednisolone was effective only for the first month, then also associated with ketogenic diet. However, at 5 months he experienced a focal status epilepticus lasting 24 h. MD was not present in this patient at the age of 15 months (12).

No brain MRI abnormalities were detected in these patients.

### HPCA

*HPCA* mutations cause autosomal recessive dystonia (MIM # 224500).

This gene encodes for the neuron-specific calcium-binding protein HPCA. HPCA is widely expressed in the brain, particularly in the hippocampal pyramidal neurons and in medium spiny neurons of the striatum, where it is likely to modulate dopamine signaling, influencing the activity of potassium and calcium channels (2, 53).

*HPCA* deficiency was initially associated to isolated dystonia (53), but subsequent reports expanded the clinical phenotype, describing patients presenting with variable combinations of seizures, developmental delay, intellectual disability, psychiatric symptoms, and dysphagia.

In two cases, a mild neurodevelopmental delay was noticed. Six individuals with ID are reported. When available, the neuropsychological assessment revealed prominent issues in verbal comprehension and/or verbal fluency.

Psychiatric symptoms such as severe anxiety and mild to severe depression are described.

Dystonia remains the core feature and is reported in seven out of nine described individuals, although other hyperkinetic MDs such as chorea and athetosis have been reported (53). Dystonia usually appears in childhood, mainly affecting the trunk, arms, and face. Orofacial dyskinesia and dysarthria have been reported, more frequently in patients with the c.182C>T p. (Ala61Val) variant. The age of onset of the disease ranges from eight months to eight years (52, 55). Therapeutic management of MD is not systematically reported. In some patients, treatment was not required, while in the two treated patients dopaminergic, anticholinergic, and antiepileptic drugs (valproate and clonazepam) were all equally ineffective.

Infantile seizures were reported in two individuals, associated with the c.225C>A, (p.Asn75Lys) pathogenetic variant (55). A further patient suffered from two episodes of febrile seizures (55). No epileptiform abnormalities were seen in most patients.

Brain MRI was normal in all patients.

## Discussion

Here, we reviewed the clinical phenotypes and mutational spectrum associated with genetic disorders affecting the GPCRs–cAMP signaling pathway and having epilepsy and movement disorder in their clinical spectrum. Common pathophysiology has been proposed but not fully investigated, as well as the degree of clinical overlap, which is the object of this work.

A complex hyperkinetic MD with or without paroxysmal exacerbations seems to be the clinical signature of the whole group. Dominant mutations in *GNAO1, GNB1*, and *PDE2A* have been associated to a complex early-onset neurological disorder characterized by a variable association of hyperkinetic MD, epilepsy, and developmental delay generally evolving into intellectual disability (23, 42, 56). *GNAL* and *ADCY5* genes have not been associated with epilepsy so far. A homozygous non-sense variant affecting the *GPR88* gene has been reported in four sisters of a single family with childhood-onset chorea and psychomotor retardation. Since then, no additional patients carrying LOF variants in this gene have been identified, raising concerns on the effective relevance of *GPR88* as a disease-causing gene. Dyskinetic storms or minor paroxysmal choreo-dystonic spells, baseline dystonia and/or chorea, prominent cranial involvement leading to dysarthria and dysphagia, orofacial dyskinesia, axial hypotonia, and severe impairment of postural development characterize the MD phenotype of these conditions. Susceptibility to a wide range of triggers and severe paroxysmal exacerbations evolving into status dystonicus are typical (*ADCY5, GNAO1, GNB1, PDE2A*). Febrile and upon awakening exacerbations of movement disorder have been described for *GNAO1, ADCY5, PDE2A*, and GNB1 (12, 42, 48, 57). Other typical triggers are emotional stress, sudden movements, and sudden sensorial stimuli. Benzodiazepines (clonazepam, lorazepam, midazolam) are useful for controlling or aborting paroxysmal episodes (*GNAO1, GNB1*), while tetrabenazine can partially control the baseline hyperkinetic MD and prevent MD exacerbations (*GNAO1, ADCY5)*. Neuromodulation (GPi-DBS or pallidotomy) has been successful in controlling paroxysmal exacerbations and evolution into status dystonicus in GNAO1, GNB1, and PDE2A-related MDs.

Epilepsy can be prominent in *GNB1* and *GNAO1* encephalopathy, while it is anecdotical in individuals with *HPCA, PDE2A*, and *PDE10A* pathogenic variants.

DEE, described in the literature as Ohtahara syndrome, infantile spasms, or EIFMS can be the epileptic presentation of *GNAO1, GNB1, and PDE2A*. Onset with tonic seizures or infantile spasms with EEG patterns of burst suppression or hypsarrhythmia has been described for *GNAO1* and *GNB1*.

These infantile forms are usually drug-resistant. Childhood-onset presentations with focal (motor and non-motor) and/or generalized seizures are usually milder. Febrile status epilepticus and febrile seizures are typical of *GNB1* and *GNAO1*.

Global developmental delay and subsequent moderate to severe intellectual disability are observed in almost all patients with *GNB1, GNAO1, PDE2A*, and *HPCA* variants. Severe motor impairment with limited postural control, absence of independent walking, and absent speech are more frequent in *GNB1* and *GNAO1* encephalopathy. Patients with *PDE10A* and *ADCY5* disorders can be cognitively normal.

Language impairment can be profound, especially in *GNAO1* and *GNB1* encephalopathy, and it is not clear if it is related to the prominent oromandibular distribution of movement disorders, or if it depends on cognitive impairment with or without oral dyspraxia. Unfortunately, this differentiation is not detailed in the actual literature where the language impairment is reported early in life, and in most cases, it is not clear if MD with oromandibular involvement coexisted at that time. MRI findings are usually nonspecific and include delayed or abnormal myelination (*GNB1, GNAO1*), cortical atrophy (*GNAO1, GNB1*), increased ventricular spaces (*GNB1*), and abnormalities of basal ganglia (*GNAO1, PDE10A*).

The presence of a core of very distinctive and shared features suggests that genetic disorders affecting the GPCRs–cAMP pathway can recognize, at least in part, the same pathophysiology.

Conversely, the different distribution and differential expression of clinical manifestations among the different disorders suggest that differentiated biological substrates, neuronal populations, or brain areas can be involved in pathophysiology and phenomenology.

*GNB1* mutations have been found in different hematological neoplasm cell lines and are thought to increase the activation of AKT/ERK/mTOR signaling (58). This finding may account for the presence of cutaneous mastocytosis and acute lymphoblastic leukemia in *GNB1* patients and, possibly, even for the presence of polymicrogyria (23).

Interestingly, a recent study in human blood neoplasms has also found *GNAO1* mutations to activate AKT/ERK/mTOR signaling (59). It is thought that the hyperactivation of mTOR pathway, as seen in other disorders such as sclerosis tuberous, *PTEN*, and *GATOR1* complex deficiency (*including DEPDC, NPRL2, and NPRL3* deficiencies), may cause epilepsy through the alteration of normal neural networks (60). The *GNAO1* variant associated to mTOR hyperactivation is the R209C variant (58). Interestingly, we found the same variant to be associated with epilepsy in 50% of the cases. Preclinical studies are needed to understand the pathophysiology of epilepsy in GNAO1 and GNB1 deficiency and to clarify the role of the mTOR signaling pathway activation.

**Figure 5:**
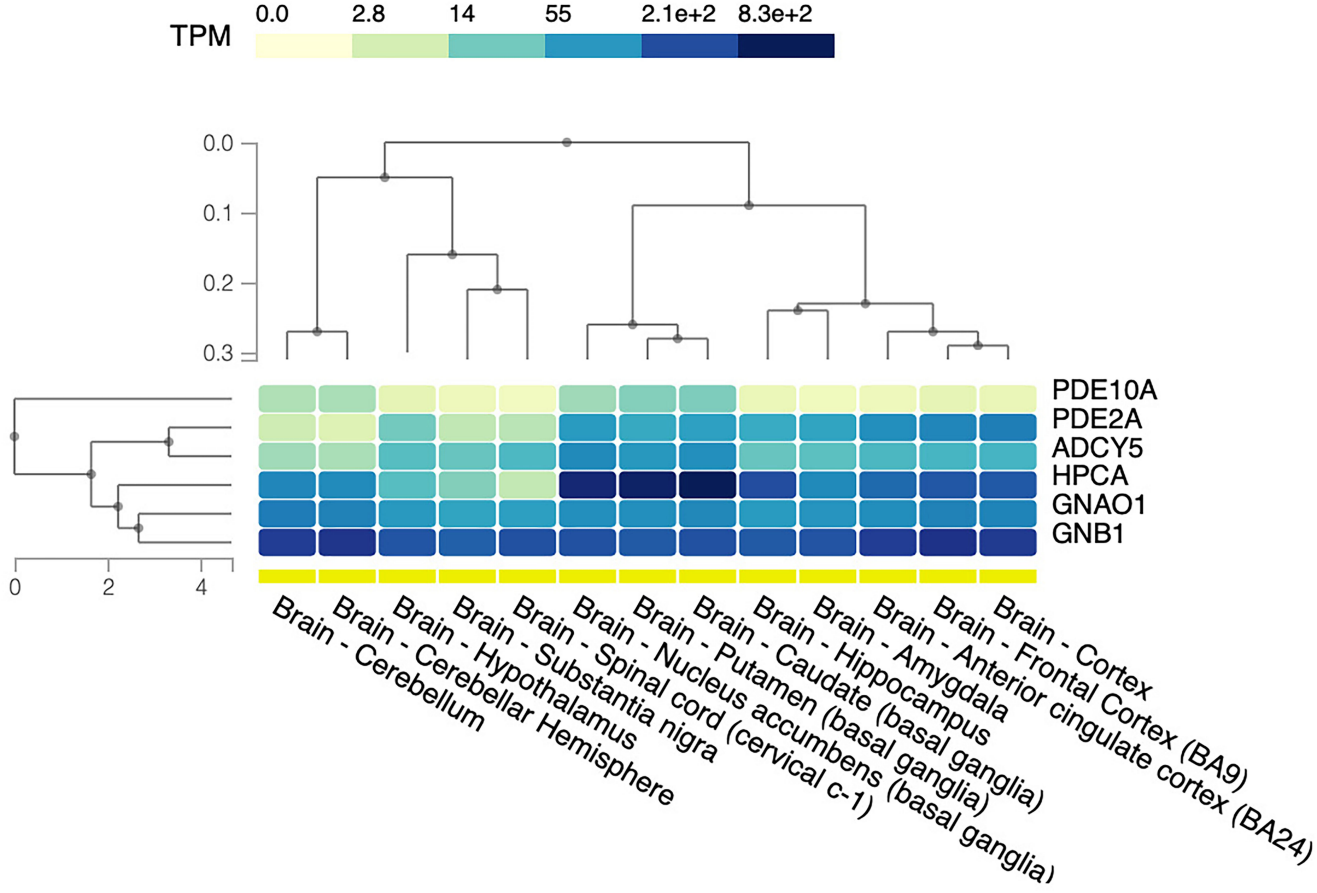
Brain tissue expression of protein related to GPCRs–cAMP signaling. The heat map shows relative gene expression indicated as transcripts per kilobase per million mapped reads (TPM) based on the color scale. The data and image were obtained from: https://www.gtexportal.org, the GTEx Portal on 29/04/22. BA, Brodmann area.

Coherently with previous observations, a different brain tissue expression of these proteins has been reported. GNAO1, GNB1, and PDE2A are ubiquitously expressed in the brain, with high levels in cortical areas, while the highest HPCA, ADCY5, and PDE10A expressions are within putamen, caudate, and nucleus accumbens [data from the Genotype-Tissue Expression (GTEx) project (https://gtexportal.org/home/)] (61) (Figure 5).

Transduction of dopaminergic and adenosine inputs from heterotrimeric GPCRs activates a cascade contributing, together with other molecular actors (e.g., PDEs), to cAMP production. The generated cAMP propagates downstream signaling *via* specific cAMP-binding proteins (e.g., cAMP-dependent kinases, transcription factors, or ion transporters). This pathway, ubiquitously expressed in the central nervous system, seems to be particularly relevant for the proper functioning of MSNs of the direct and indirect pathways, and therefore for postural control, initiation of voluntary movements, prevention of unwanted movements, and motor learning (2, 5, 15).

Specifically, cAMP levels finely regulate the activity of protein such as the cAMP-regulated phosphoprotein molecular mass 32 (DARPP-32) and the cAMP-response element-binding protein (CREB). These proteins are thought to play an important role by mediating the dopaminergic neuromodulatory effects on GABAergic transmission and regulating the long-term synaptic plasticity and neuronal growth at MSNs level (62). Thus, through an altered basal ganglia activity, altered cAMP levels may underpin movement disorders such as dystonia, chorea, and parkinsonism.

The contribution of this pathway to neurodevelopment has been less explored. The possible individuation of common mechanisms rather than mechanisms specific to certain disorders deserves further studies.

Here, we reviewed the motor, epileptic, and neurodevelopmental phenotype of genetic neurological disorders affecting GPCRs–cAMP signaling pathway. This group of disorders presents with a highly recognizable clinical phenotype with distinctive movement disorder, epileptic, and neurodevelopmental features. While no biomarker is available for this group of potentially life-threatening disorders, the existence of distinctive clinical features prompting diagnostic suspicion and early detection has relevant implications for patient management.

## Author contributions

SG and LP: data collection, first draft writing. VL and SM: critical review. MD, KB, and MN: data collection. All authors contributed to the article and approved the submitted version.

## Conflict of interest

The authors declare that the research was conducted in the absence of any commercial or financial relationships that could be construed as a potential conflict of interest.

## Publisher's note

All claims expressed in this article are solely those of the authors and do not necessarily represent those of their affiliated organizations, or those of the publisher, the editors and the reviewers. Any product that may be evaluated in this article, or claim that may be made by its manufacturer, is not guaranteed or endorsed by the publisher.
