# Supplementary material for: Motor, epileptic, and developmental phenotypes in genetic disorders affecting G protein coupled receptors-cAMP signaling
Source: Front Neurol. 2022 Aug 8;13:886751. doi: 10.3389/fneur.2022.886751 (PMC9393484; doi:10.3389/fneur.2022.886751)
Supplement: Supplementary file 1 [file Data_Sheet_1.docx]

**Supplemental Table 1. Clinical phenotypes of GNAO1 recurrent variants**

|  | c.118G>A | c.118G>C | c.119G>A | c.607G>A | c.625C>T | c.626 G>A | c.626G>T | c.644G>A | c.680C>T | c.692A>G | c.709G > A | c.724-8G>A | c.736G>A | c.818A>T | c.836T>A |
| --- | --- | --- | --- | --- | --- | --- | --- | --- | --- | --- | --- | --- | --- | --- | --- |
|  | p.Gly40Arg | p.Gly40Arg | p.Gly40Glu | p.Gly203Arg | p.Arg209Cys | p.Arg209His | p.Arg209Leu | p.Cys215Tyr | p.Ala227Val | p.Tyr231Cys | p.Glu237Lys |  | p.Glu246Lys | p.Asp273Va | p.Ile279Asn |
| N. of cases | 2 | 3 | 2 | 11 | 12 | 10 | 2 | 3 | 2 | 3 | 6 | 4 | 13 | 2 | 3 |
| DD/ID | 2 | 3 | 2 | 11 | 12 | 10 | 2 | 1 | 2 | 3 | 6 | 3 | 13 | 2 | 3 |
| Dysphagia/Tube feeding | 0 | 1 | 1 | 5 | 6 | 4 | 1 | 1 | 0 | 0 | 2 | 1 | 8 | 1 | 3 |
| Epilepsy | 2 | 3 | 2 | 11 | 6 | 0 | 0 | 0 | 2 | 3 | 0 | 0 | 1 | 1 | 3 |
| Epilepsy onset  Neonatal onset  Infantile onset  Childhood onset  Adolescent onset  ND | 2  0  0  0  0 | 0  3  0  0  0 | 2  0  0  0  0 | 8  2  1  0  0 | 0  2  4  0  0 | / | / | / | 0  1  1  0  0 | 2  1  0  0  0 | / | / | 1  0  0  0  0 | 1  0  0  0  0 | 3  0  0  0  0 |
| Seizure types  Spasms  Behavioural Arrest  Generalized  Focal  Unspecified | 0  0  2  0  0 | 3  0  2  2  0 | 2  0  2  0  0 | 3  1  6  6  0 | 0  0  4  3  0 | / | / | / | 0  1  0  2  0 | 0  2  3  0  0 | / | / | 0  0  0  1  1 | 0  0  1  0  0 | 3  1  0  2  0 |
| EEG  Burst suppression  Hypsarrhythmia Slow background  Focal disch.  Multifocal disch.  Gen. disch.  Not specified | 0  0  0  0  0  2  0 | 1  0  1  2  0  2  0 | 0  0  1  1  1  1  0 | 0  0  1  2  1  6  2 | 0  0  0  4  1  0  0 | 0  0  0  2  0  0  0 | 0  0  0  0  0  0  0 | 0  0  0  0  0  0  0 | 0  0  1  0  1  1  0 | 1  0  1  0  0  2  0 | 0  0  0  0  0  0  0 | 0  0  0  2  0  0  0 | 0  0  0  0  1  0  0 | 0  0  0  0  0  0  0 | 2  0  1  0  0  2  0 |
| AED  No drugs needed  Controlled by AED  Drug-resistant  ND | 0  0  0  2 | 0  1  1  1 | 0  0  0  2 | 0  4  7  0 | 0  4  1  1 | / | / | / | 0  1  1  0 | 0  1  1  1 | / | / | 0  1  0  0 | 0  0  1  1 | 0  0  2  1 |
| Spasticity | 0 | 0 | 0 | 0 | 4 | 2 | 0 | 0 | 0 | 2 | 0 | 0 | 2 | 0 | 0 |
| Movement disorder | 0 | 1 | 1 | 10 | 11 | 10 | 2 | 3 | 0 | 2 | 6 | 4 | 12 | 2 | 2 |
| MD onset  Neonatal onset  Infantile onset  Childhood onset  Adolescent onset  Unspecified | / | 0  1  0  0  0 | / | 0  6  0  0  4 | 0  6  3  0  2 | 0  9  1  0  0 | 0  2  0  0  0 | 0  0  1  0  2 | / | 0  1  0  0  1 | 1  2  2  0  1 | 0  2  1  0  1 | 0  9  3  0  0 | 0  1  0  0  1 | 0  0  0  0  2 |
| MD type  Chorea/Ath  Ballismus Dystonia  Myoclonus  Ataxia  Bradykinesia  Unspecified | / | 0  0  0  0  1  0  0 | 0  0  1  0  0  0  0 | 6  2  7  0  0  0  7 | 7  4  8  0  1  1  6 | 8  1  7  2  3  0  2 | 1  0  2  1  0  0  1 | 0  0  3  3  0  0  1 | / | 0  0  2  0  0  0  1 | 3  3  5  1  0  2  4 | 1  0  3  1  2  1  1 | 10  5  7  0  1  1  5 | 0  1  1  0  0  0  0 | 2  0  0  0  0  0  2 |
| MD life threating events/Status dystonicus | / | 0 | 0 | 4 | 9 | 5 | 1 | 0 | / | 0 | 3 | 0 | 5 | 0 | 0 |
| Drug  No drug needed  Controlled  Not controlled  ND | / | 0  0  0  1 | 0  0  0  1 | 0  1  8  1 | 0  0  9  2 | 0  0  5  5 | 0  0  1  1 | 0  1  0  2 | / | 0  0  0  2 | 1  0  3  2 | 1  3  0  0 | 0  1  6  5 | 0  0  1  1 | 0  0  0  2 |
| DBS | / | 0 | 0 | 0 | 4 | 3 | 1 | 1 | / | 0 | 3 | 0 | 3 | 0 | 0 |
| Note: | / | / | / | / | / | / | / | / | / | / | / | / | / | / | / |

**Supplemental table 2. Clinical phenotypes of GNB1 recurrent variants**

|  | c.158 G>A | c.229G>A | c.233 A>G | c.239 T>A | c.239 T>C | c.284 T>C | c.287 G>T | c.301 A>G | c.353 A>G |
| --- | --- | --- | --- | --- | --- | --- | --- | --- | --- |
|  | p.Gly53Glu | p.Gly77Ser | p.Lys78Arg | p.Ile80Asn | p.Ile80Thr | p.Leu95Pro | p.Arg96Leu | p.Met101Val | p.Asp118Gly |
| N. of cases | 2 | 1 (+1*) | 2 | 2 | 13 | 5 | 3 | 2 | 4 |
| DD/ID | 2 | 1 | 2 | 2 | 13 | 5 | 3 | 2 | 4 |
| Dysphagia/Tube feeding | 0 | 0 | 2 | 2 | 5 | 4 | 0 | 1 | 1 |
| Epilepsy | 0 | 0 | 1 | 2 | 6 | 3 | 2 | 2 | 0 |
| Epilepsy onset  Neonatal onset  Infantile onset  Childhood onset  Adolescent onset  ND | / | / | 0  1  0  0  0 | 0  1  1  0  0 | 0  2  4  0  0 | 0  3  0  0  0 | 0  0  0  0  2 | 0  1  1  0  0 | / |
| Seizure types  Spasms  Behavioural Arrest  Generalized  Focal  Unspecified | / | / | 1  0  0  0  0 | 0  0  1  1  0 | 1  0  5  2  0 | 2  0  1  0  0 | 0  0  0  0  2 | 1  0  3  1  0 | / |
| EEG  Burst suppression  Hypsarrhythmia  Slow background  Focal disch.  Multifocal disch.  Gen. disch.  Not specified  ND | 0  0  0  0  0  0  0  0 | 0  0  1  0  0  0  0  0 | 0  0  1  0  1  0  0  0 | 0  0  1  0  1  1  0  0 | 0  0  1  1  3  2  0  0 | 0  0  0  0  1  2  1  0 | 0  0  0  0  0  0  0  2 | 0  1  1  1  1  1  0  0 | 0  0  0  0  0  0  0  0 |
| AED  No drugs needed  Controlled by AED  Drug-resistant  ND | 0  0  0  0 | 0  0  0  0 | 0  1  0  0 | 0  0  0  2 | 0  2  3  1 | 0  1  1  1 | 0  0  0  2 | 0  0  2  0 | 0  0  0  0 |
| Spasticity | 1 | 0 | 0 | 0 | 4 | 1 | 0 | 0 | 0 |
| Movement disorder | 0 | 0 | 0 | 1 | 6 | 2 | 0 | 0 | 2 |
| MD onset  Neonatal onset  Infantile onset  Childhood onset  Adolescent onset  Unspecified | / | / | / | 0  0  0  0  1 | 0  1  0  0  0 | 0  1  0  0  1 | / | / | 0  0  0  0  2 |
| MD type  Chorea/Ath  Ballismus  Dystonia  Myoclonus  Ataxia  Bradykinesia  Unspecified | / | / | / | 0  0  1  0  0  0  0 | 1  0  5  0  0  1  1 | 0  0  2  0  0  0  0 | / | / | 0  0  2  1  0  0  0 |
| MD life threating events/Status dystonicus | / | / | / | 0 | 0 | 1 | 0 | 0 | 0 |
| Drug  No drug needed  Controlled  Not controlled  ND | / | / | / | 0  0  0  1 | 0  0  0  6 | 0  0  1  1 | 0  0  0  0 | 0  0  0  0 | 0  0  1  1 |
| DBS | / | / | / | 0 | 0 | 0 | 0 | 0 | 0 |
| Note: | / | / | / | / | Nystagmus, Mastocytosis | Cleft palate, Nystagmus | / | / | / |

*= only autism spectrum disorder has been reported. No other information available
